# Variant analysis of the sporozoite surface antigen gene reveals that asymptomatic cattle from wildlife-livestock interface areas in northern Tanzania harbour buffalo-derived *T. parva*

**DOI:** 10.1007/s00436-020-06902-1

**Published:** 2020-10-03

**Authors:** Micky M. Mwamuye, David Odongo, Yvette Kazungu, Fatuma Kindoro, Paul Gwakisa, Richard P. Bishop, Ard M. Nijhof, Isaiah Obara

**Affiliations:** 1 Institute for Parasitology and Tropical Veterinary Medicine, Freie Universität Berlin, Robert-von-Ostertag-Str. 7-13, 14163 Berlin, Germany; 2 School of Biological Sciences, University of Nairobi, Nairobi, Kenya; 3 Genome Science Laboratory, College of Veterinary Medicine and Biomedical Sciences, Sokoine University of Agriculture, P.O. Box 3019, Morogoro, Tanzania; 4 Department of Veterinary Microbiology & Pathology, Washington State University, Pullman, WA USA

**Keywords:** *Theileria parva*, p67, Tp2, Antigen diversity, Cape Buffalo, Live vaccine

## Abstract

Buffalo-derived *Theileria parva* can ‘break through’ the immunity induced by the infection and treatment vaccination method (ITM) in cattle. However, no such ‘breakthroughs’ have been reported in northern Tanzania where there has been long and widespread ITM use in pastoralist cattle, and the Cape buffalo (*Syncerus caffer*) is also present. We studied the exposure of vaccinated and unvaccinated cattle in northern Tanzania to buffalo-derived *T. parva* using p67 gene polymorphisms and compared this to its distribution in vaccinated cattle exposed to buffalo-derived *T. parva* in central Kenya, where vaccine ‘breakthroughs’ have been reported. Additionally, we analysed the CD8+ T cell target antigen Tp2 for positive selection. Our results showed that 10% of the p67 sequences from Tanzanian cattle (*n* = 39) had a buffalo type p67 (allele 4), an allele that is rare among East African isolates studied so far. The percentage of buffalo-derived p67 alleles observed in Kenyan cattle comprised 19% of the parasites (*n* = 36), with two different p67 alleles (2 and 3) of presumptive buffalo origin. The Tp2 protein was generally conserved with only three Tp2 variants from Tanzania (*n* = 33) and five from Kenya (*n* = 40). Two Tanzanian Tp2 variants and two Kenyan Tp2 variants were identical to variants present in the trivalent Muguga vaccine. Tp2 evolutionary analysis did not show evidence for positive selection within previously mapped epitope coding sites. The p67 data indicates that some ITM-vaccinated cattle are protected against disease induced by a buffalo-derived *T. parva* challenge in northern Tanzania and suggests that the parasite genotype may represent one factor explaining this.

**Electronic supplementary material:**

The online version of this article (10.1007/s00436-020-06902-1) contains supplementary material, which is available to authorized users.

## Introduction

*Theileria parva* is a tick-transmitted protozoan tick-borne haemo-pathogen which causes East Coast fever (ECF), a frequently lethal and economically important disease of cattle in Eastern, Central and Southern sub-Saharan Africa (Norval et al. 1991). ECF has been conservatively estimated to be responsible for US$ 300 million of loss annually and kills approximately 1 million cattle per year (Mukhebi et al. 1992). The disease is most severe in exotic taurine cattle in the absence of control but has also been demonstrated, particularly in Tanzania and Kenya, to induce high mortality in zebu calves in resource-poor pastoralist systems (Di Giulio et al. 2009; Thumbi et al. 2013). After nearly a century of acaricide use in the management of tick-borne diseases including ECF, the development of resistance by ticks, environmental contamination and food safety concerns resulting from toxic residues are rendering the approach unsustainable in the medium term (George et al. 2004). Chemotherapy of ECF is usually ineffective in the absence of early diagnosis which is often impractical in field situations (Matovelo et al. 2003).

The ability to grind up whole infected *R. appendiculatus* vector ticks and cryopreserve *T. parva* sporozoites that can be inoculated into cattle simultaneously treated with a long-acting formulation of oxytetracyline was critical to the development of the infection and treatment method (ITM) of live vaccination (Cunningham et al. 1973; Radley et al. 1975). This approach remains the only practical way of inducing immunity against *T. parva* in cattle, as there are currently no subunit vaccines that provide protection equal or superior to ITM. Early experiments demonstrated that a panel of three stocks of *T. parva* provided broader protection against heterologous challenge than any single stock (Cunningham et al. 1974). These cross-protection experiments resulted in the development of the trivalent Muguga cocktail version of ITM which comprises the Muguga, Serengeti-transformed and Kiambu 5 stocks of *T. parva* (Radley et al. 1975). The logistical and policy constraints related to production and delivery that initially constrained ITM deployment and the technical developments that have increased ITM adoption have been the subject of a recent review (Bishop et al. 2020).

One aspect of *T. parva* epidemiology pertinent to ITM efficacy is the presence of a wildlife reservoir population of the parasite in the Cape buffalo (*Syncerus caffer*). Exposure of cattle to buffalo-derived *T. parva* results in Corridor disease, a frequently lethal clinical syndrome characterised by low levels of schizonts, the parasite’s intra-lymphocytic stage and scanty piroplasm parasitaemia (Irvin and Mwamachi 1983; Jura and Losos 1980). These pathological features are distinct from ECF caused by *T. parva* that is transmissible between cattle by ticks, which tends to be associated with a high piroplasm parasitaemia. Importantly, studies have shown that ITM-vaccinated cattle can be susceptible to challenge with parasites from buffalo. The earliest study of this kind involved keeping ITM-immunised cattle in paddocks together with buffalo (Radley et al. 1979). The conclusion of the research was that *T. parva* parasites from ticks fed on buffalo could sometimes ‘break through’ the immunity induced by ITM. While both buffalo and cattle *T. parva* strains have identical life cycles, evidence suggests that the ECF causing *T. parva* is better adapted to cattle and is thought to represent a restricted subset of the overall *T. parva* population (Pelle et al. 2011; Sitt et al. 2019). Two recent attempts to immunise cattle against buffalo-derived *T. parva* in field trials by grazing ITM-immunised cattle adjacent to buffalo in ranches in central Kenya both resulted in vaccinated animals contracting severe disease (Bishop et al. 2015; Sitt et al. 2015).

No comparable studies have been undertaken to monitor the effects of ITM use in field situations where cattle are exposed to *T. parva* originating from buffalo in the other East African countries, including Uganda and Tanzania where several wildlife-livestock interface areas also exist. Of the very limited ITM vaccinations that have been performed in cross-bred or taurine cattle in Uganda, none has been in areas where cattle are exposed to field challenge by buffalo-derived parasites (Nsubuga-Mutaka 1999). By contrast, the first successful large-scale deployment of the original trivalent Muguga cocktail version of ITM, without apparent ‘breakthrough’ infections, has been in Maasai pastoralist systems in northern Tanzania, where there has been long-term co-grazing between cattle and Cape buffalo (Di Giulio et al. 2009). Understanding the reason for absence of breakthrough infections in northern Tanzania will be important for assessing the probability of future success of the cocktail in other wildlife-livestock interface regions.

The explanation advanced for the failure of ITM to induce cross-protection against locally circulating *T. parva* populations in wildlife-livestock interface areas is that the cocktail contains only a very small proportion of the diversity within the *T. parva* gene pool in Kenya (Hemmink et al. 2016; Pelle et al. 2011; Sitt et al. 2015). Cape buffalo is known to be infected with multiple *T. parva* genotypes (Oura et al. 2011) and harbours a much greater diversity of the parasite based on the analysis of genes encoding schizont antigens that are the focus of ongoing recombinant vaccine trials (Pelle et al. 2011). The extent to which the gene pools of buffalo and cattle *T. parva* populations are separate in Tanzania is not known. In addition, there is as yet no evidence that vaccinated cattle immune to ECF in the Maasai pastoralist systems in northern Tanzania are being challenged by *T. parva* transmitted by ticks that have fed on buffalo. To address these issues, we have sampled pastoralist cattle from an area in northern Tanzania that is contiguous with the parks, reserves and conservation areas, where there has been long-term co-grazing between the cattle and buffalo.

Transmission of *T. parva* from buffalo to cattle was sought by examining the major sporozoite surface antigens (p67) alleles that have been shown in recent studies to be presumptive of *T. parva* originating from buffalo (Nene et al. 1996; Obara et al. 2015; Sibeko et al. 2010; Sitt et al. 2019)*.* Four p67 alleles have been described based on the presence or absence of 129- and 174-bp deletions within the p67 gene central region (Nene et al. 1996; Sibeko et al. 2010). Within East Africa, *T. parva* parasites that are transmissible between cattle by ticks have been frequently observed to carry the p67 allele type 1 which is relatively conserved and is characterised by a 129 bp deletion in the central region as exemplified by the Muguga strain of *T. parva* (Nene et al. 1996). By contrast, the *T. parva* carrying the more heterogeneous p67 allele types 2–4 is known to originate from buffalo and has been shown to be associated with Corridor disease in livestock-wildlife interface areas, where cattle are exposed to infective ticks that have fed on infected African Cape buffalo (Mukolwe et al. 2020; Obara et al. 2015; Sibeko et al. 2010; Sitt et al. 2019). Recently however, a type 1 allele also associated with buffalo *T. parva* from South Africa but differing from the East Africa cattle type 1 p67 alleles by amino acid changes in the areas flanking the deletion and the epitope regions has been described (Mukolwe et al. 2020). In addition to assessing the level of exposure of Tanzanian pastoralist cattle to buffalo type *T. parva* based on p67 gene polymorphism, we also determined how similar the p67 alleles at the interface areas in Tanzania were to alleles carried by buffalo-associated *T. parva* strains in Kenya. Additionally to provide an initial indication of how variable the genes encoding *T. parva* schizont antigens are in northern Tanzania, we have also studied the molecular evolution of a *T. parva* candidate subunit vaccine antigen (Tp2) containing epitopes known to be recognised by immune African cattle cytotoxic T lymphocytes (Akoolo et al. 2008). For comparison, we also analysed blood samples from central Kenya obtained from clinically reacting cattle that exhibited the typical signs of Corridor disease in an ITM controlled trial (Bishop et al. 2015).

## Materials and methods

### Sample collection and processing

**Fig. 1:**
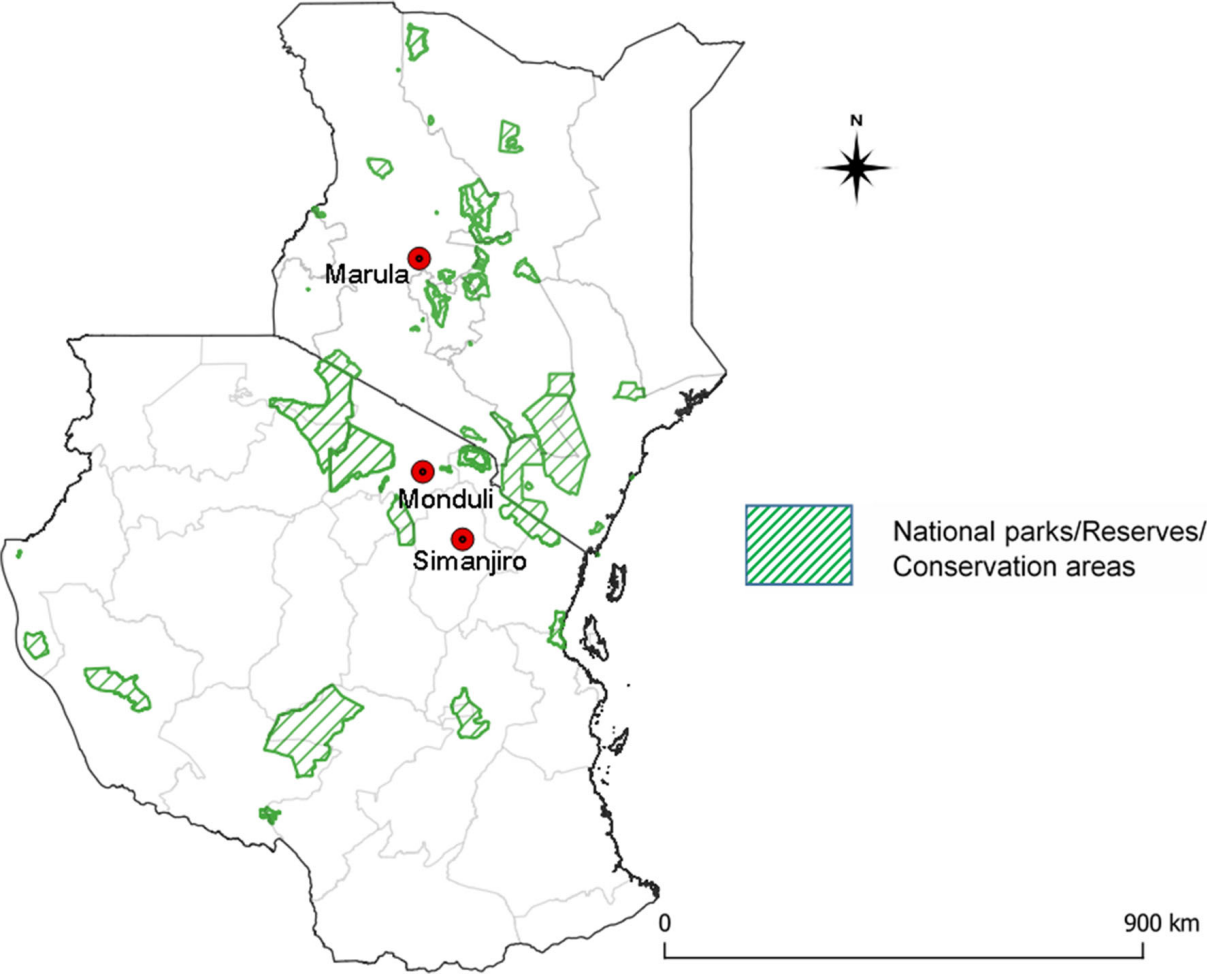
Map showing the origin of blood samples used in this study from in Kenya and Tanzania

The Tanzanian study areas comprised the Monduli and the Simanjiro plains in northern Tanzania (Fig. 1), which are areas of intensified wild-domestic animal interaction due to proximity to game reserves and national parks. These regions are predominantly inhabited by pastoralist Maasai communities who largely depend on livestock for subsistence and represent areas with wide deployment of the ITM vaccine against ECF in Tanzania. Five villages in Simanjiro (Loiborsoit, Terrat, Emboret, Narakawo and Sukuro) were selected based on their proximity to national parks. Consent from local authorities and individual farmers was obtained after explaining the outline and relevance of the study. Certified veterinary personnel collected blood from asymptomatic cattle that included both non-vaccinated (*n* = 104) and vaccinated (*n =* 56) cattle that were differentiated on the basis of ear tag numbers that indicated year of vaccination with confirmation by respective farmers. Additionally, buffalos (*n =* 22) were sampled from the Serengeti National Park with the involvement of Tanzania Wildlife Research Institute (TAWIRI). Blood was collected in 10-ml EDTA vacutainer tubes that were temporarily maintained in ice-cooled boxes while on transit to the laboratory for further processing at the Genome Science laboratory, College of Veterinary Medicine and Biomedical Sciences, Sokoine University of Agriculture.

The archived blood samples from Kenya (*n* = 100) included in this study were originally collected during an ITM efficacy trial in the Marula area of Kenya (Bishop et al. 2015). Briefly, *T. parva* naive cattle were vaccinated using the Muguga cocktail version of ITM and exposed to natural challenge by grazing adjacent to buffalo. DNA from frozen whole blood samples was isolated using NucleoSpin Blood Mini kit for DNA from blood (Macherey-Nagel) according to manufacturer’s protocol.

### Amplification, cloning and sequencing of the *T. parva* p67 and Tp2 genes

Previously designed primers targeting a 838-bp fragment of the p67 gene (IL 6133: 5′- ACAAACACAATCCCAAGTTC-3′; IL 7922: 5′-CCTTTACTACGTTGGCG-3′) and a 525-bp fragment of the Tp2 gene (forward primer: 5′-ATGAAATTGGCCGCCAGATTA-3′; reverse primer: 5′-CTATGAAGTGCCGGAGGCTTC-3′) were used to amplify the respective genes by PCR (Nene et al. 1996; Pelle et al. 2011). The PCR amplification reaction mixture consisted of 5 × High-Fidelity (HF) Phusion buffer (ThermoFisher Scientific), 200-μM dNTPs (Biozym Scientific GmbH), 0.5 μM of both forward and reverse primers, 0.02 U/μl HF Phusion DNA polymerase and 2 μl of template DNA. The reactions were topped up to a final volume of 25 μl using sterile PCR-grade water (Carl Roth GmbH, Karlsruhe). Non-template controls and in-house *T. parva* schizont DNA extracted from infected cell lines were implemented for quality control. The thermal profile included an initial 30-s step at 98 °C, 40 cycles of 98 °C for 30 s, an annealing step at 58 °C (p67) or 63.6 °C (Tp2) for 40 s and elongation at 72 °C for 1 min, followed by a final extension step for 10 min at 72 °C. Successful amplification was resolved by gel electrophoresis with GRGreen (Excellgene, Monthey, Switzerland) as a DNA stain. Successfully amplified products were column-purified using GeneJET PCR Purification Kit (ThermoFisher Scientific) prior to bidirectional Sanger sequencing (LGC Genomics, Berlin).

Since buffalos are frequently infected with multiple strains of *T. parva*, Tp2 and p67 amplicons from buffalo samples were cloned using the StrataClone Blunt PCR Cloning Kit (Agilent Technologies, USA). Plasmids were purified using GenUP™ Plasmid Kit (biotechrabbit GmbH, Germany) and evaluated by *Eco*RI restriction digestion (ThermoFisher Scientific). Plasmids bearing inserts with the expected size were sequenced using standard M13 primers by LGC Genomics.

### Sequence analyses and alignments

Forward and reverse sequence reads were assembled de novo, edited and translated into their predicted protein sequences using Geneious Prime 2019.2 (Biomatters, Ltd.). A 29-bp intron was deleted prior to the translation of p67 sequences into protein. All p67 sequences were categorised based on the absence/presence of a 43 aa deletion initially described by Nene et al. (1996). The buffalo-derived p67 alleles were further classified into allele types 2, 3 and 4 according to a previously published classification scheme (Sibeko et al. 2010). For reference, we used four published sequences with Accession Numbers XP_763305, AAB0673, AFU34359 and AFU34364 representing alleles 1–4, respectively (Supplementary Fig. 1). All p67 sequences missing the 43 aa were designated as cattle-derived *T. parva* strains. All unique sequences were queried against the online GenBank database using the Basic Local Alignment Search Tool (BLAST) for similarity matches with other published sequences. Nucleotide and amino acid sequence alignments were generated using MAFFT V.7 and MUSCLE, respectively, and percent pairwise identity matrices were generated in Geneious Prime 2019.2 software.

### Selection analysis in the Tp2 coding sequence

As available evidence shows that long-term immunity induced by ITM is largely mediated by CD8^+^ T cell responses, we focused our analysis for historical selection on the Tp2 coding sequence. We considered the Tanzania cattle and buffalo Tp2 sequences as one regional *T. parva* population; hence the Tp2 sequences were grouped into 2 populations (Tanzanian and Kenyan Tp2), and separate codon optimised alignments were created using MUSCLE codon in MEGAx (Kumar et al. 2018). Due to sequence length differences resulting from quality trimming of the non-cloned cattle sequences, the aligned matrices were truncated to equal lengths of 483 bp spanning the six mapped epitope region to create gapless alignments. The likelihood scores for 88 different nucleotide substitution models were computed for each aligned sequence matrix, and the best-fitting model was selected based on the Akaike information criterion (AIC) using the jModeltest2 program (Darriba et al. 2012). Using the selected model, maximum likelihood (ML) phylogenies were inferred using PAUP version 4 (Swofford 2003), and nonsynonymous to synonymous nucleotide substitution rates were estimated using the CodeML tool of PAML package (Yang 2007) implemented in the EasyCodeML visual selection analysis tool (Gao et al. 2019). Molecular evolution site models (M1a, M2a, M7, M8) were evaluated, and maximum likelihood inferences of positive selection based on AIC to compare model fit (M1a: neutral against M2a: positive selection) and M7 (β) vs. M8 (β and ω) were performed. Positively selected sites were identified based on Bayes empirical Bayes (BEB) posterior probability calculations (Yang et al. 2005).

## Results

A total of 160 and 100 cattle samples were processed from northern Tanzania and Central Kenya, respectively. However, we successfully amplified 39 and 36 sequences for the *T. parva* p67 gene and 33 and 40 sequences for the *T. parva* Tp2 gene from Tanzania and Kenya, respectively. Out of 22 analysed buffalo samples, only four generated PCR amplicons from which ten unique p67 and six Tp2 cloned sequences were obtained.

### Tanzania pastoralist cattle are exposed to buffalo-associated *T. parva* strains

From the 39 *T. parva* p67 sequences from pastoralist cattle northern Tanzania, we observed a high level of sequence conservation with 85% of these sequences (*n* = 33) being identical at the nucleotide level and classed as cattle-derived on the basis of the 43 aa deletion in the central region of the gene. BLAST analysis using the 33 sequences as input revealed 100% identity to AVT43014, a p67 allele that was isolated from cattle co-grazing with buffalo in a game conservancy in central Kenya (Sitt et al. 2019). Two other unique p67 sequences also exhibited > 99% identity to AVT43014 at the protein level (Table 1). Interestingly, four animals had p67 sequences that did not contain the 43 aa deletion and were therefore categorised as being of buffalo origin. Further analysis revealed that these four sequences also did not have the additional 174 bp deletion used to distinguish allele 3 from allele type 4. These four sequences translated into two unique amino acid sequences (MT365019 and MT365020) both of which exhibited > 95% identity to the AFU34364 isolated from a South African buffalo (Sibeko et al. 2010).

**Table 1:** P67 allele type and GenBank sequence similarity search for the unique p67 predicted proteins in the study samples

	Cattle type p67	Buffalo type p67			NCBI BLASTp hits		
	Allele 1	Allele 2	Allele 3	Allele 4	Identity	Accession no.	Isolate origin
Tanzania							
Buffalo							
MT344093 [1]		+			100.00%	AVT43026	Cattle
MT344094 [1]			+		100.00%	AVT43035	Buffalo
MT344095 [1]			+		100.00%	AVT43037	Cattle
MT344096 [1]			+		100.00%	AAB06703	Buffalo
MT344097 [1]		+			100.00%	AVT43025	Cattle
MT344098 [1]			+		99.63%	AVT43048	Cattle
MT344099 [1]			+		99.25%	AVT43037	Cattle
MT344100 [1]			+		98.14%	AVT43050	Cattle
MT344101 [1]		+			99.25%	AVT43025	Cattle
MT344102 [1]		+			95.15%	AVT43037	Cattle
Cattle							
MT365016 [33]	+				100.00%	AVT43014	Cattle
MT365017 [1]	+				99.31%	AVT43014	Cattle
MT365018 [1]	+				99.64%	AVT43014	Cattle
MT365019 [2]				+	95.97%	AFU34364	Buffalo
MT365020 [2]				+	96.31%	AFU34364	Buffalo
Kenya							
Cattle							
MT365005 [1]		+			100.00%	AVT43025	Cattle
MT365006 [1]		+			99.70%	AVT43025	Cattle
MT365007 [1]		+			99.69%	AVT43025	Cattle
MT365009 [29]	+				100.00%	AVT43014	Cattle
MT365011 [1]			+		100.00%	AVT43047	Buffalo
MT365012 [1]			+		99.61%	AVT43031	Buffalo
MT365013 [1]			+		100.00%	AVT43037	Cattle
MT365014 [1]			+		99.56%	AVT43040	Cattle

The number enclosed [ ] represents the number of alleles

These findings were comparable to the results of the analysis that we conducted on the samples from the interface area in Kenya. In particular, of the 36 samples typed for p67 allele, 81% (*n* = 29) were also 100% identical to the cattle type p67 allele, AVT43014, that was isolated from cattle co-grazing with buffalo at the Ol Pejeta Conservancy in central Kenya (Sitt et al. 2019). Similarly, only a minority of the animals typed (*n* = 7) were classified as being of buffalo origin (Table 1).

Out of the 22 analysed buffalo samples, only four buffalo samples generated PCR amplicons from which ten unique p67 cloned sequences were obtained comprising allele types 2 and 3 (Table 1).

### p67 epitope polymorphisms

Six antibody epitopes have been mapped on the p67 protein, two of which are closely juxtaposed in the central region of the protein (Nene et al. 1999). The first of these two epitopes within the central region (TmM12 epitope) was conserved in all sequences categorised as cattle type (allele 1) or buffalo type (allele 2) except for one sequence from Kenya cattle (MT365007) which had a single substitution (TKEEVPPADLSDQV**L**) (Table 2). This amino acid substitution has been observed among cattle type alleles from South Africa in a recent study (Mukolwe et al. 2020). As shown in Table 2, two buffalo p67 type 3 alleles had 7 substitutions in this epitope that have previously been reported in Kenya and South Africa (Sibeko et al. 2010; Sitt et al. 2019), while the remaining buffalo type 3 and 4 alleles had 5 substitutions (Table 2). AR22.7 is the second epitope in the central region (LQPGKTS) and our analysis shows that it was conserved in all cattle type 1 alleles and a single buffalo type 2 allele (MT44097), while all remaining buffalo type 2 alleles in this study had a single substitution (L**P**PGKTS) when compared with the *T. parva* Muguga stock. The AR22.7 epitope orthologous sequences in both buffalo types 3 and 4 were identical but were conserved at only three positions **L**KN**G**R**T**D with respect to the *T. parva* Muguga reference stock (Table 2).

**Table 2:** Epitope polymorphisms within predicted p67 amino acid sequences in this study

		P67 antibody epitope	
Allele type	Accession number	TpM12	AR22.7
1	XP_763305 (Graham et al. 2005)	TKEEVPPADLSDQVP	LQPGKTS
	MT365016–018^a^	**- - - - - - - - - - - - - - -**	**- - - - - - -**
	MT365009^c^	**- - - - - - - - - - - - - - -**	**- - - - - - -**
2	AAB06703 (Nene et al. 1996)	**- - - - - - - - - - - - - - -**	**- - - - - - -**
	MT344097^b^	**- - - - - - - - - - - - - - -**	**- - - - - - -**
	MT344093^b^	**- - - - - - - - - - - - - - -**	**-** P **- - - - -**
	MT344101–102^b^	**- - - - - - - - - - - - - - -**	**-** P **- - - - -**
	MT365005–006^c^	**- - - - - - - - - - - - - - -**	**-** P **- - - - -**
	MT365007^c^	**- - - - - - - - - - - - - -** L	**-** P **- - - - -**
3	AFU34359 (Sibeko et al. 2010)	**- - - - - - -** KSD **-** ESEQ	**-** KN **-**R **-**D
	MT344098–099 ^b^	**- - - - - - -** KSD **-** ESEQ	**-** KN **-**R **-**D
	MT365011^c^	**- - - - - - - -** SS **- -** SEQ	**-** KN **-**R **-**D
	MT344100^b^	**- - - - - - - -** SS **- -** SEQ	**-** KN **-**R **-**D
	MT344094–096^b^	**- - - - - - - -** SS **- -** SEQ	**-** KN **-**R **-**D
	MT365012–014^c^	**- - - - - - - -** SS **- -** SEQ	**-** KN **-**R **-**D
4	AFU34364 (Sibeko et al. 2010)	**-** N **- - - - - - - - - -** SEQ	**-** KN **-**R **-**D
	MT365019–020^a^	**- - - - - - - -** SG **- -** SEQ	**-** KN **-**R **-**D

^a^Sequences amplified from Tanzanian cattle

^b^Sequences amplified from Tanzanian buffalo

^c^Sequences amplified from Kenyan cattle

(-) Dashes represent amino acid residues that are identical to the reference sequence

### Sequence diversity in the Tp2 candidate vaccine antigen in the interface areas in Tanzania and Kenya samples

**Fig. 2:**
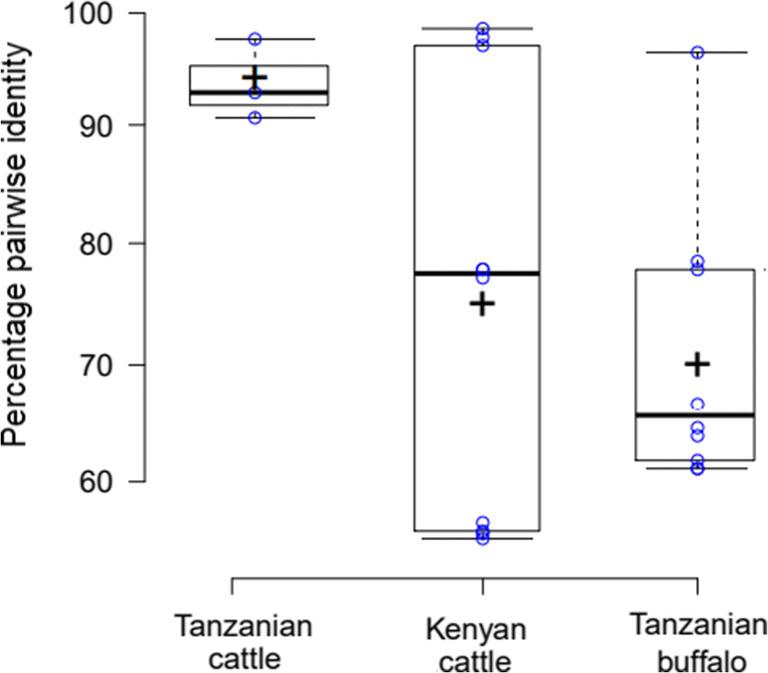
Percentage pairwise amino acid identity in the unique Tp2 sequences among the different sample groups. Box limits indicate the 25th and 75th percentiles; median is represented by thick lines across the boxes; crosses represent sample means; whiskers extend 1.5 times the interquartile range from the 25th and 75th percentiles. The matrix used for this plot is available in Supplementary Table 1

Tp2 is a *T. parva* candidate subunit vaccine antigen containing epitopes recognised by cytotoxic T lymphocytes from immune African cattle (Akoolo et al. 2008). We amplified the gene encoding Tp2 from the 33 African indigenous zebu cattle sampled in northern Tanzania. Collapsing of identical reads revealed a total of 13 unique Tp2 nucleotide sequences from the Tanzanian zebu population, three of which had substitutions that were nonsynonymous at the amino acid level. Two out of the three unique Tp2 sequences have previously been described and one was novel (MT334677). The previously described Tp2 protein sequences were 100% identical to Tp2 variants present within the *T. parva* Muguga/Serengeti (MT334672) and Kiambu-5 strain variants (MT334671). Percentage pairwise comparisons of the unique Tp2 predicted proteins (Fig. 2) showed a higher level of similarity within Tanzania cattle samples with an average amino acid similarity of 95.10% (± 2.81 SD).

Our analysis showed a similar level of polymorphisms in the Tp2 antigen gene in the Marula cattle-buffalo interface area in Kenya. In particular, 9 unique Tp2 nucleotide sequences were identified from the 40 cattle sampled in Kenya. These translated into 5 protein variants (Accession nos. MT334662–MT334666). Two variants (MT334665 and MT334666) were identical to *T. parva* Muguga/Serengeti and *T. parva* Kiambu-5 Tp2 antigens, respectively, while one protein variant (MT334663) was identical to a Tp2 variant (AFC18340) in a *T. parva* isolate that originated from a Kenyan buffalo. Additionally, this latter protein variant (MT334663) was identical to two Tanzania buffalo *T. parva* Tp2 clones (MT334658 and MT334659) obtained from the current study. Two novel Tp2 protein variants (MT334664 and MT334662) from Kenya are reported in this study. As shown in Fig. 2, percentage pairwise comparisons of these unique Tp2 from Kenya revealed a mean pairwise amino acid identity of 75.46% (±18.28 SD).

It is noteworthy that of the 6 Tanzanian buffalos that we typed for Tp2, all the animals carried unique Tp2 nucleotide sequences and only two were synonymous at the amino acid level. The five unique Tanzanian buffalo Tp2 sequences have been deposited in GenBank with accession numbers: MT334656–MT334658 and MT334660–MT334661. As expected, most of the diversity in the Tp2 antigen gene was seen in isolates of buffalo origin, with a mean pairwise percent identity of 70% (Fig. 2).

### Novel Tp2 epitope variants

**Fig. 3:**
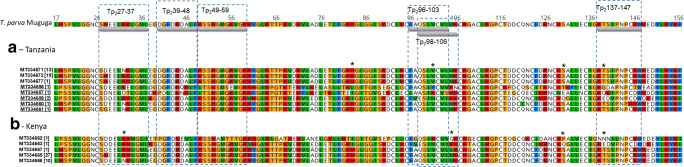
Tp2 predicted amino acid sequences from this study. The number enclosed [ ] represents the number of alleles. Underlined accession numbers are buffalo-derived *T. parva* Tp2 sequences. Thickened lines underneath the reference sequence show respective epitope mapped positions on the *T. parva* (Muguga) stock. (*) denotes codon suggested to be under positive selection based on Bayes empirical Bayes (BEB) analysis (Table 4) (Yang et al. 2005)

The analysis of the six mapped Tp2 epitopes within the predicted proteins showed sequences obtained from Tanzania cattle to be the most conserved with only two epitopes, Tp_2_27-37 and Tp_2_137-147, being polymorphic. Epitope Tp_2_27-37 had two variants (S**H**EEL**K**KLGML and S**D**EEL**N**KLGML), which are closest to Muguga/Serengeti and Kiambu-5, respectively (Fig. 1, Table 3). Epitope Tp_2_137-147 also had two variants (KTS**I**PNPCKW and KTS**V**PNPCKW), the latter of which is a novel variant from this study while the former is conserved in the three Muguga cocktail vaccine stocks (Pelle et al., 2011). In contrast, 2–5 epitope variants were identified in all the Kenyan Tp2 epitope sequences, while the Tanzanian buffalo Tp2 sequences had at least 3–5 variants per epitope (Table 3). Further, one novel variant (S**G**EELNKLGML) within epitope Tp_2_27-37 and two variants (K**Q**KIPNP**C**DW and K**T**SIPNP**R**KW) within epitope Tp_2_137-147 were identified in the buffalo Tp2 sequences (Fig. 3, Table 3).

**Table 3:** Epitope variants identified in this study based on the predicted Tp2 protein translations

	Epitope variant					
	Tp2_27-37_	Tp2_39-48_	Tp2_49-59_	Tp2_96-103_	Tp2_98-106_	Tp2_137-147_
Reference	SHEELKKLGML	DGFDRDALF	KSSHGMGKVGK	FAQSLVCVL	QSLVCVLMK	KTSIPNPCKW
Tanzania						
Cattle						
	-D---N-----					---**V**------^*^
Buffalo						
	-D----E----	-----QR--	LT-KSMSE--R	----IY--V	--IY--VKN	-GDA----T-
	-DN--DT-G-L	PDL-KNR--	LT------I-R	L-A-IK--S	A-IK--SHH	-QK-----D-^*^
	-*DD*--*R*-----		----------R	-VE-IM--I	E-IM--IK-	-EDV----D-
	-D---NK----					-------R--^*^
	-G---N-----^*^					
Kenya						
Cattle						
	-DD----M--I	P---KEV--	---KA-TTT--	-G--IK--V	--IK--VQ-	NNN-L-----
	-*DD*--*R*-----		----------R	-VE-IM--I	E-IM--IK-	-EDV----D-
	-*D*---------					
	-H---------					
	-D---N-----					

(-) Dashes represent amino acid residues that are identical to the reference sequence. Only epitopes with variant residues are displayed

(^*^) Depicts novel variants identified in this study

Italicised variants are described in Kerario et al. (2019) while all other variants have previously been identified in Pelle et al. (2011)

### Selective pressure acting on the Tp2 antigen gene

For the Tanzanian Tp2 dataset, Akaike weights provided more support for the positive selection model M8 when compared with the null, neutral model M7. The proportion of sites determined to be evolving under positive selection was 0.03024 (3.02%) with ω = 4.03249. Four codon sites were suggested under BEB analysis to be positively selected, two of which were within the known epitopes but none of the four codons had statistically significant support for positive selection (*p* > 0.05). For the Kenyan Tp2 dataset, application of both model pairs, M1 with M2 and M7 with M8, supported evidence for positive selection (Table 4). According to the M8 model, 5 codon sites (ω = 35.4258) were under positive selection which represented 2.64% of the analysed codons. However, only one site T159 exhibited statistical support for selection (*p* < 0.01), but this was located outside the known mapped epitopes (Table 4).

**Table 4:** Evaluation of model fit by the Akaike information criterion (AIC), model parameter estimates and positively selected sites under the M8 site model using Bayes empirical Bayes (BEB) analysis

	Model fit				Estimate of parameters				Positive sites
Tp2 sample	Model	K	LnL	AIC	***ω***	p0	p1	p2	
Tanzania	M8	44	− 1714.80	3517.60	4.03249	0.9680		0.03020	83 H 0.519, 101 V 0.557, 130 S 0.925, 138 T 0.762
	M7	42	− 1716.84	3517.69					Not allowed
Kenya	M2a	48	− 1309.83	2715.65	37.9162	0.6986	0.2763	0.02511	
	M1a	46	− 1315.46	2722.92		0.6627	0.3373		Not allowed
	M8	48	− 1309.15	2714.30	35.4258	0.9736		0.02644	32 N 0.646, 105 M 0.869, 130 S 0.824, 138 T 0.645, 159 T 0.995**
	M7	46	− 1315.70	2723.41					Not allowed

K denotes number of estimated parameters; LnL denotes maximised log likelihoods; p0 (purifying), p1(neutral) and p2 (positive) indicate the proportion of codons belonging to each site class, while ω represents the dN/dS for the positive selection site class only. (**) denotes > 99% posterior probabilities of positive selection. The codon numbers are identified with reference to the *T. parva* Muguga Tp2 antigen

## Discussion

The infection and treatment method of immunisation against *T. parva* uses stabilates produced from parasites that are transmissible between cattle. The ability of *T. parva* derived from Cape buffalo to ‘break through’ the immunity induced by ITM has been documented in multiple studies in Kenya (Bishop et al. 2015; Radley et al. 1979; Sitt et al. 2015). Vaccinated cattle may be susceptible to challenge with parasites from buffalo because the immunity induced by ITM is partially strain-specific, and the diversity of *T. parva* genotypes is greater in Cape buffalo than in cattle as revealed by genome sequencing (Hayashida et al. 2012), variation within antigen encoding genes (Pelle et al. 2011) and analyses of the variable number of tandem repeats (VNTR) (Oura et al. 2011). There have as yet been no studies of ITM effectiveness, or parasite population genetic diversity in areas where buffalo interface closely with cattle in Tanzania, and the question of genetic similarity between parasite populations in cattle and buffalo has not yet been addressed. Interestingly, in contrast to Kenya, there has not been any observation of ‘breakthrough’ in ITM-immunised cattle by buffalo-derived parasites in the field in northern Tanzania, where there has been widespread adoption of ITM on a large scale especially among the pastoralist communities in northern Tanzania (Di Giulio et al. 2009). Our present analyses represent an exploration of whether multiple buffalo-derived *T. parva* genotypes, with variable p67 sequences, are infecting cattle herds located in close proximity with buffalo in northern Tanzania.

Cattle-transmissible *T. parva* genotypes in East Africa frequently carry type 1 p67 alleles, whereas p67 alleles 2, 3 and 4 represent markers for parasites with a high probability of a recent origin from buffalo (Nene et al. 1996; Obara et al. 2015). The current study establishes that although a majority of the cattle in the northern Tanzania region are infected with cattle-transmissible *T. parva* genotypes, some of the animals are infected with *T. parva* genotypes with p67 sequences suggesting a likely recent origin from buffalo. This finding generally conforms to the distribution of p67 alleles at the interface area in Kenya that we evaluated (although the buffalo-derived p67 genotypes were different) and also to the situation reported in previous studies (Mukolwe et al. 2020; Obara et al. 2015; Sitt et al. 2019). It is also important to note that this pattern of p67 allelic polymorphism is consistent with a scenario whereby frequent transmission of parasites from buffalo to cattle occurs, but many fail to permanently establish in cattle following initial infection, because insufficient progression of buffalo-derived parasites to the tick-infective erythrocytic piroplasm stage occurs to enable onward transmission between cattle by ticks (Mbizeni et al. 2013; Morrison et al. 1989). Interestingly, our analysis showed that the cattle in northern Tanzania were infected with buffalo-derived *T. parva* genotypes classified as type 4 allele on the basis of their p67 sequences. The fact that we did not detect the buffalo-derived allele type 4 of p67in our buffalo samples may have been the result of under sampling; hence, further in-depth sampling will improve the understanding of allele distribution patterns in the region. Previous analyses had suggested that this allele, which has been found in buffalo *T. parva* in South Africa, seems to be rare within East Africa (Mukolwe et al. 2020; Obara et al. 2015; Sibeko et al. 2010; Sitt et al. 2019). By contrast, buffalo-derived *T. parva* alleles 2 and 3 genotypes which we found in our Kenyan sample dataset were also reported in previous studies (Obara et al. 2015; Sitt et al. 2019), but not observed in the Tanzanian cattle samples studied.

Recombinant versions of the *T. parva* p67 major sporozoite surface protein, combined with adjuvants, have been extensively evaluated as anti-sporozoite vaccine candidates. Needle challenges using sporozoite stabilates derived from ground up whole ticks consistently resulted in approximately 70% efficacy against either heterologous or homologous challenge in the laboratory (Bishop et al. 2003; Musoke et al. 1992). However, when cattle were exposed to natural tick challenge in the field, the levels of protection observed were only 45% (Musoke et al. 2005). Although the reason for this reduction is not understood, and such field studies are yet to be undertaken in areas where cattle interface with buffalo, p67 polymorphisms in the epitopes recognised by anti-sporozoite monoclonal antibodies are nevertheless worthy of investigation. Our analysis revealed a previously unidentified p67 epitope variant. Contrary to the complete conservation of the p67 locus in cattle-transmissible *T. parva* isolates that have been studied, we observed an amino acid substitution **L**^183^ within the Kenyan p67 sequence (accession no. MT365007) in the epitope defined by tmM12 (^169^TKEEVPPADLSDQV**P**^183^). This amino acid substitution has been observed in a buffalo-derived *T. parva* type 1 allele from South Africa in a recent study (Mukolwe et al. 2020). However, unlike the single substitution reported in this study, the South African type 1 allele carries two polymorphisms within the TmM12 epitope when compared with the *T. parva* Muguga reference stock.

It is also important to note that long-term immunity against *T. parva* has been demonstrated to be mediated by CD8+ T cells specific for schizont-infected lymphocytes (McKeever et al. 1994). *T. parva* genes encoding antigens that are the targets of this protective response in exotic taurine cattle have been identified and named Tp1-Tp8 (Graham et al. 2006), and Tp9–Tp10 (Morrison et al. 2015). Subsequent studies showed that, with the exception of Tp2, peptides derived from the Tp antigens are not recognised by immune bovine CD8+ T cells from African Zebu (*Bos indicus*) cattle (Akoolo et al. 2008). We therefore studied variation in the Tp2 epitopes and assessed the evidence for positive selection in the buffalo-associated cattle isolates from northern Tanzania and from the Marula area in central Kenya. Four novel Tp2 epitope variants were found, three of which were identified in the buffalo Tp2 sequences from Tanzania. Interestingly, one of the four variants identified from the Tanzanian cattle sample was within epitope Tp2_137-147_ (Table 2), which is an epitope known to be recognised by cytotoxic T lymphocytes (CTL) from African Zebu cattle (Akoolo et al. 2008), which are the predominant breed kept by the Maasai in northern Tanzania. Consistent with previous studies of the molecular evolution of the Tp2 antigen, our analysis did not provide evidence for enrichment of positively selected codons within the mapped Tp2 epitopes (Amzati et al. 2019; Pelle et al. 2011). However, ideally the molecular evolution of the Tp2 and other antigen genes should be addressed without the confounding issue of the potential presence of sequences derived from the ITM vaccine. ITM-vaccinated animals remain carriers of a tick-transmissible infection for up to 14 years (Gwakisa et al. 2020) and ITM genotypes have been detected in unvaccinated cattle. A carrier state of this longevity might result in vaccination contributing to homogenisation of parasite populations.

One major distinction between the cattle sampled in Kenya and those from Tanzania is that the infected Kenyan cattle exhibited a typical buffalo-derived *T. parva* clinical syndrome characterised by low schizont parasitosis and piroplasm parasitaemia, and many ultimately died (Bishop et al. 2015). By contrast, the cattle sampled from northern Tanzania were asymptomatic and presumably represented carrier animals that had survived infection. The reasons for this difference in susceptibility are unclear. One possible explanation for this observation is that there is extensive genetic exchange between buffalo-derived and cattle-derived parasites due to long-term buffalo-derived parasite tick challenge of Maasai cattle in Tanzania and that those calves that survive to adulthood are immune to local parasites. Although there is presently insufficient data to confirm this hypothesis, such genetic exchange and alteration of allelic frequencies have been demonstrated experimentally by feeding ticks on cattle co-infected with parasite clones and analysing progeny clones for evidence of recombination (Henson et al. 2012; Katzer et al. 2011).

In conclusion, the evidence of exposure of cattle in northern Tanzania to buffalo-derived *T. parva* shows the need for further investigations into parasite population genomics and the contribution to induction of immunity in adult cattle as a result of natural challenge by ticks that have fed on buffalo. It will also be important to identify *T. parva* genes encoding antigens that are recognised by bovine CD8 T cells from immune zebu and buffalo. Additionally, a study using a larger sample size that simultaneously examines the tick and the parasites present in the vector, in wildlife-cattle interface areas where there has been large-scale ITM deployment, should further illuminate the long-term effects of vaccination on *T. parva* population genomics.

## Electronic supplementary material

Supplementary Table 1 Pairwise identity matrix for the unique Tp2 sequences in this study (DOCX 14 kb)

Supplementary Fig. 1 P67 multiple sequence alignment used to generate the classification of the alleles identified in this study (DOCX 406 kb)

## References

[CR1] Akoolo L, Pellé R, Saya R, Awino E, Nyanjui J, Taracha ELN, Kanyari P, Mwangi DM, Graham SP (2008). Evaluation of the recognition of *Theileria parva* vaccine candidate antigens by cytotoxic T lymphocytes from Zebu cattle. Vet Immunol Immunopathol.

[CR2] Amzati GS, Djikeng A, Odongo DO, Nimpaye H, Sibeko KP, Muhigwa JBB, Madder M, Kirschvink N, Marcotty T (2019). Genetic and antigenic variation of the bovine tick-borne pathogen *Theileria parva* in the Great Lakes region of Central Africa. Parasit Vectors.

[CR3] Bishop R, Nene V, Staeyert J, Rowlands J, Nyanjui J, Osaso J, Morzaria S, Musoke A (2003). Immunity to East Coast fever in cattle induced by a polypeptide fragment of the major surface coat protein of *Theileria parva* sporozoites. Vaccine.

[CR4] Bishop RP, Hemmink JD, Morrison WI, Weir W, Toye PG, Sitt T, Spooner PR, Musoke AJ, Skilton RA, Odongo DO (2015). The African buffalo parasite *Theileria* sp. (buffalo) can infect and immortalize cattle leukocytes and encodes divergent orthologues of *Theileria parva* antigen genes. Int J Parasitol Parasites Wildl.

[CR5] Bishop RP, Odongo D, Ahmed J, Mwamuye M, Fry LM, Knowles DP, Nanteza A, Lubega G, Gwakisa P, Clausen PH, Obara I (2020). A review of recent research on *Theileria parva*: implications for the infection and treatment vaccination method for control of East Coast fever. Transbound Emerg Dis.

[CR6] Cunningham MP, Brown CGD, Burridge MJ, Irvin AD, Kirimi IM, Purnell RE, Radley DE, Wagner GG (1974). Theileriosis: the exposure of immunized cattle in a *Theileria lawrencei* enzootic area. Trop Anim Health Prod.

[CR7] Cunningham MP, Joyner LP, Brown CGD, Purnell RE, Bailey KP (1973). Infection of cattle with East Coast fever by inoculation of the infective stage of *Theileria parva* harvested from the tick vector *Rhipicephalus appendiculatus*. Bull epizoot Dis Afr.

[CR8] Darriba D, Taboada GL, Doallo R, Posada D (2012). jModelTest 2: more models, new heuristics and parallel computing. Nat Methods.

[CR9] Di Giulio G, Lynen G, Morzaria S, Oura C, Bishop R (2009). Live immunization against East Coast fever--current status. Trends Parasitol.

[CR10] Gao F, Chen C, Arab DA, Du Z, He Y, Ho SYW (2019). EasyCodeML: a visual tool for analysis of selection using CodeML. Ecol Evol.

[CR11] George JE, Pound JM, Davey RB (2004). Chemical control of ticks on cattle and the resistance of these parasites to acaricides. Parasitology.

[CR12] Graham SP, Pelle R, Honda Y, Mwangi DM, Tonukari NJ, Yamage M, Glew EJ, de Villiers EP, Shah T, Bishop R, Abuya E, Awino E, Gachanja J, Luyai AE, Mbwika F, Muthiani AM, Ndegwa DM, Njahira M, Nyanjui JK, Onono FO, Osaso J, Saya RM, Wildmann C, Fraser CM, Maudlin I, Gardner MJ, Morzaria SP, Loosmore S, Gilbert SC, Audonnet JC, van der Bruggen P, Nene V, Taracha ELN (2006). *Theileria parva* candidate vaccine antigens recognized by immune bovine cytotoxic T lymphocytes. Proc Natl Acad Sci U S A.

[CR13] Gwakisa P, Kindoro F, Mwega E, Kimera S, Obara I, Ahmed J, Clausen PH, Bishop R (2020). Monitoring vaccinated cattle for induction and longevity of persistent tick-transmissible infection: implications for wider deployment of live vaccination against East Coast fever in Tanzania. Transbound Emerg Dis.

[CR14] Hayashida K et al (2012) Comparative genome analysis of three eukaryotic parasites with differing abilities to transform leukocytes reveals key mediators of *Theileria*-induced leukocyte transformation. mBio 3(5):e00204-12. 10.1128/mBio.00204-12 10.1128/mBio.00204-12 PMC3445966 22951932

[CR15] Hemmink JD, Weir W, MacHugh ND, Graham SP, Patel E, Paxton E, Shiels B, Toye PG, Morrison WI, Pelle R (2016). Limited genetic and antigenic diversity within parasite isolates used in a live vaccine against *Theileria parva*. Int J Parasitol.

[CR16] Henson S, Bishop RP, Morzaria S, Spooner PR, Pelle R, Poveda L, Ebeling M, Küng E, Certa U, Daubenberger CA, Qi W (2012). High-resolution genotyping and mapping of recombination and gene conversion in the protozoan *Theileria parva* using whole genome sequencing. BMC Genomics.

[CR17] Irvin AD, Mwamachi DM (1983). Clinical and diagnostic features of East Coast fever (*Theileria parva*) infection of cattle. Vet Rec.

[CR18] Jura WGZ, Losos GJ (1980). A comparative study of the diseases in cattle caused by *Theileria lawrencei* and *Theileria parva*. 1. Clinical signs and parasitological observations. Vet Parasitol.

[CR19] Katzer F, Lizundia R, Ngugi D, Blake D, McKeever D (2011). Construction of a genetic map for *Theileria parva*: identification of hotspots of recombination. Int J Parasitol.

[CR20] Kumar S, Stecher G, Li M, Knyaz C, Tamura K (2018). MEGA X: Molecular evolutionary genetics analysis across computing platforms. Mol Biol Evol.

[CR21] Matovelo JA, et al. (2003) Induction of acquired immunity in pastoral zebu cattle against East Coast fever after natural infection by early diagnosis and early treatment Int J Appl Res Vet Med 1(2)

[CR22] Mbizeni S, Potgieter FT, Troskie C, Mans BJ, Penzhorn BL, Latif AA (2013). Field and laboratory studies on Corridor disease (*Theileria parva* infection) in cattle population at the livestock/game interface of uPhongolo-Mkuze area, South Africa. Ticks Tick Borne Dis.

[CR23] McKeever DJ (1994). Adoptive transfer of immunity to *Theileria parva* in the CD8+ fraction of responding efferent lymph. Proc Natl Acad Sci U S A.

[CR24] Morrison WI, Connelley T, Hemmink JD, MacHugh ND (2015). Understanding the basis of parasite strain-restricted immunity to *Theileria parva*. Annu Rev Anim Biosci.

[CR25] Morrison WI, Goddeeris BM, Brown WC, Baldwin CL, Teale AJ (1989). *Theileria parva* in cattle: characterization of infected lymphocytes and the immune responses they provoke. Vet Immunol Immunopathol.

[CR26] Mukhebi AW, Perry BD, Kruska R (1992). Estimated economics of theileriosis control in Africa. Prev Vet Med.

[CR27] Mukolwe L, Odongo D, Byaruhanga C, Snyman L, Sibeko-Matjila K (2020). Analysis of p67 allelic sequences reveals a subtype of allele type 1 unique to buffalo-derived *Theileria parva* parasites from southern Africa. PLoS One.

[CR28] Musoke A, Morzaria S, Nkonge C, Jones E, Nene V (1992). A recombinant sporozoite surface antigen of *Theileria parva* induces protection in cattle. Proc Natl Acad Sci U S A.

[CR29] Musoke A, Rowlands J, Nene V, Nyanjui J, Katende J, Spooner P, Mwaura S, Odongo D, Nkonge C, Mbogo S, Bishop R, Morzaria S (2005). Subunit vaccine based on the p67 major surface protein of *Theileria parva* sporozoites reduces severity of infection derived from field tick challenge. Vaccine.

[CR30] Nene V, Gobright E, Bishop R, Morzaria S, Musoke A (1999). Linear peptide specificity of bovine antibody responses to p67 of *Theileria parva* and sequence diversity of sporozoite-neutralizing epitopes: implications for a vaccine. Infect Immun.

[CR31] Nene V, Musoke A, Gobright E, Morzaria S (1996). Conservation of the sporozoite p67 vaccine antigen in cattle-derived *Theileria parva* stocks with different cross-immunity profiles. Infect Immun.

[CR32] Norval RA, Lawrence JA, Young AS, Perry BD, Dolan TT, Scott J (1991). *Theileria parva*: influence of vector, parasite and host relationships on the epidemiology of theileriosis in southern Africa. Parasitology.

[CR33] Nsubuga-Mutaka R, Morzaria S, Williamson S (1999). ECF Immunisation in Uganda. Live vaccines for *Theileria parva*: deployment in Eastern, Central and Southern Africa.

[CR34] Obara I, Ulrike S, Musoke T, Spooner PR, Jabbar A, Odongo D, Kemp S, Silva JC, Bishop RP (2015). Molecular evolution of a central region containing B cell epitopes in the gene encoding the p67 sporozoite antigen within a field population of *Theileria parva*. Parasitol Res.

[CR35] Oura CA, Tait A, Asiimwe B, Lubega GW, Weir W (2011). Haemoparasite prevalence and *Theileria parva* strain diversity in Cape buffalo *(Syncerus caffer*) in Uganda. Vet Parasitol.

[CR36] Pelle R, Graham SP, Njahira MN, Osaso J, Saya RM, Odongo DO, Toye PG, Spooner PR, Musoke AJ, Mwangi DM, Taracha ELN, Morrison WI, Weir W, Silva JC, Bishop RP (2011). Two *Theileria parva* CD8 T cell antigen genes are more variable in buffalo than cattle parasites, but differ in pattern of sequence diversity. PLoS One.

[CR37] Radley DE (1975). East coast fever: 3. Chemoprophylactic immunization of cattle using oxytetracycline and a combination of theilerial strains. Vet Parasitol.

[CR38] Radley DE, Young AS, Grootenhuis JG, Cunningham MP, Dolan TT, Morzaria SP (1979). Further studies on the immunization of cattle against *Theileria lawrencei* by infection and chemoprophylaxis. Vet Parasitol.

[CR39] Sibeko KP, Geysen D, Oosthuizen MC, Matthee CA, Troskie M, Potgieter FT, Coetzer JAW, Collins NE (2010). Four p67 alleles identified in South African *Theileria parva* field samples. Vet Parasitol.

[CR40] Sitt T, Henson S, Morrison WI, Toye P (2019). Similar levels of diversity in the gene encoding the p67 sporozoite surface protein of *Theileria parva* are observed in blood samples from buffalo and cattle naturally infected from buffalo. Vet Parasitol.

[CR41] Sitt T, Poole EJ, Ndambuki G, Mwaura S, Njoroge T, Omondi GP, Mutinda M, Mathenge J, Prettejohn G, Morrison WI, Toye P (2015). Exposure of vaccinated and naive cattle to natural challenge from buffalo-derived *Theileria parva*. Int J Parasitol Parasites Wildl.

[CR42] Swofford DL (2003) PAUP*. Phylogenetic analysis using parsimony (*and other methods). Version 4. Sinauer Associates, Sunderland, Massachusetts

[CR43] Thumbi SM (2013). Parasite co-infections show synergistic and antagonistic interactions on growth performance of East African zebu cattle under one year. Parasitology.

[CR44] Yang Z (2007). PAML 4: phylogenetic analysis by maximum likelihood. Mol Biol Evol.

[CR45] Yang Z, Wong WS, Nielsen R (2005). Bayes empirical Bayes inference of amino acid sites under positive selection. Mol Biol Evol.

