# Supplementary material for: Variant analysis of the sporozoite surface antigen gene reveals that asymptomatic cattle from wildlife-livestock interface areas in northern Tanzania harbour buffalo-derived T. parva
Source: Parasitol Res. 2020 Oct 3;119(11):3817–28. doi: 10.1007/s00436-020-06902-1 (PMC7578158; doi:10.1007/s00436-020-06902-1)
Supplement: Supplementary file 1 — Pairwise identity matrix for the unique Tp2 sequences in this study (DOCX 14 kb) [file 436_2020_6902_MOESM1_ESM.docx]

**Supplementary Table 1.** Pairwise identity matrix for the unique Tp2 sequences in this study

| Cattle_TZ | Cattle_KE | Buffalo_TZ |
| --- | --- | --- |
| 94.44 | 56.36 | 78.74 |
| 92.68 | 57.14 | 78.16 |
| 98.18 | 56.55 | 65.52 |
|  | 56.02 | 60.92 |
|  | 78.18 | 97.13 |
|  | 77.58 | 63.22 |
|  | 78.18 | 60.92 |
|  | 97.62 | 63.79 |
|  | 98.19 | 61.49 |
|  | 98.8 | 67.24 |
|  |  |  |
|  |  |  |
|  |  |  |
|  |  |  |
|  |  |  |
|  |  |  |
|  |  |  |
|  |  |  |
|  |  |  |
|  |  |  |
|  |  |  |
|  |  |  |
|  |  |  |
|  |  |  |
|  |  |  |
|  |  |  |
|  |  |  |
|  |  |  |
