# Supplementary material for: Variant analysis of the sporozoite surface antigen gene reveals that asymptomatic cattle from wildlife-livestock interface areas in northern Tanzania harbour buffalo-derived T. parva
Source: Parasitol Res. 2020 Oct 3;119(11):3817–28. doi: 10.1007/s00436-020-06902-1 (PMC7578158; doi:10.1007/s00436-020-06902-1)

**Supplementary Fig. 1.** P67 multiple sequence alignment used to generate the classification of the alleles identified in this study


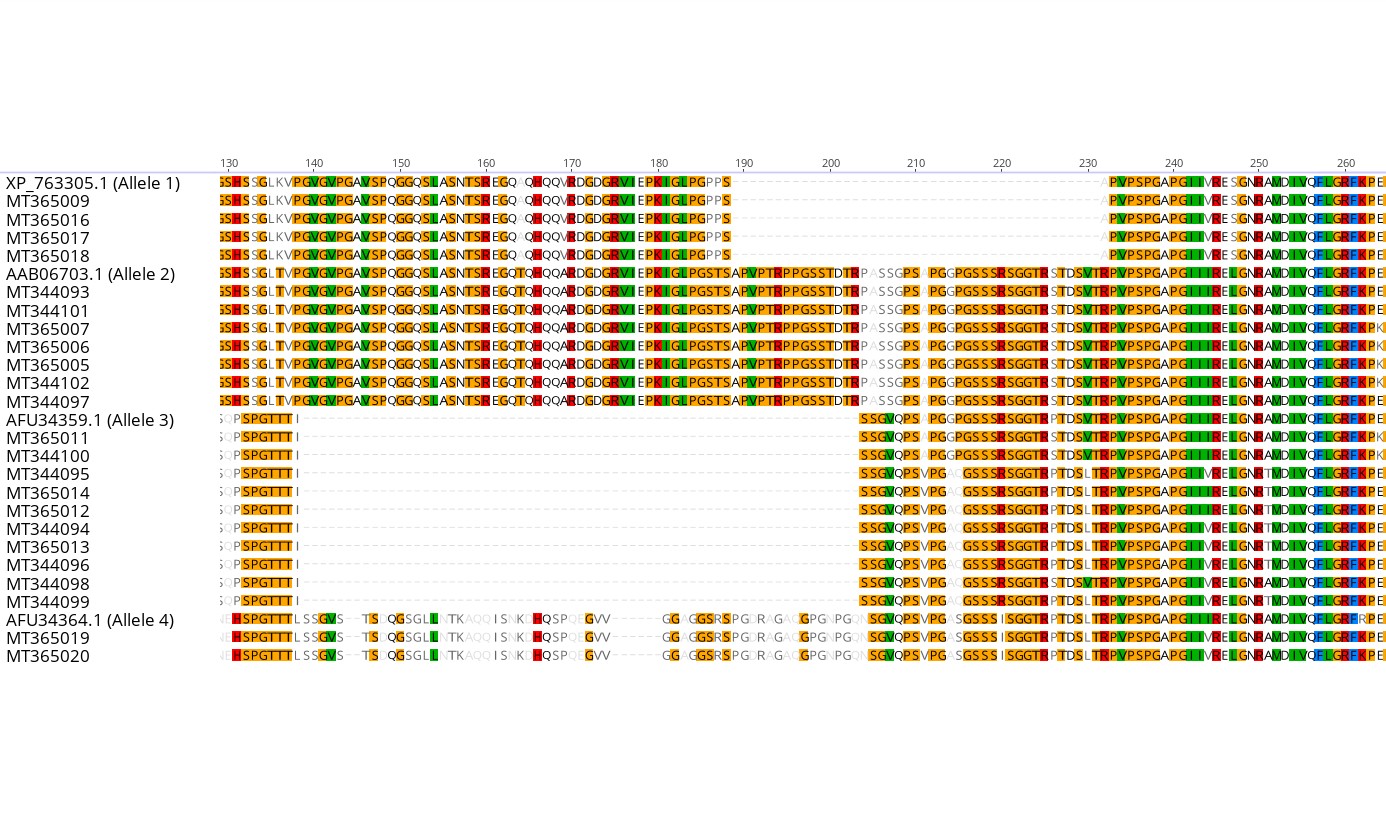

Supplement: Supplementary file 2 — P67 multiple sequence alignment used to generate the classification of the alleles identified in this study (DOCX 406 kb) [file 436_2020_6902_MOESM2_ESM.docx]
